# Orphan GPCRs in diabetes mellitus: metabolic control, inflammation, and drug development

**DOI:** 10.3389/fendo.2026.1916297

**Published:** 2026-08-19

**Authors:** Sureka Chandrabose, Vikraman Muniyandi, Ayman Geddawy, Ramesh Thiyagarajan, Akshaya Murugesan, Meenakshisundaram Kandhavelu

**Affiliations:** 1 Department of Basic Medical Sciences, College of Medicine, Prince Sattam Bin Abdulaziz University, Al-Kharj, Saudi Arabia; 2 Molecular Signaling Group, Faculty of Medicine and Health Technology, Tampere University, Tampere, Finland

**Keywords:** diabetes mellitus, inflammation, obesity, orphan GPCRs, repurposed drugs

## Abstract

Diabetes mellitus (diabetes) is a major health burden with the highest mortality rate, affecting an estimated 537 million adults worldwide. The treatment of diabetes remains complex owing to insulin resistance, β-cell dysfunction, metabolic inflammation, and progressive organ damage. While G-protein-coupled receptors (GPCRs) selectively suppress many pathological diseases, the functional characteristics of orphan GPCRs (oGPCRs) remain unexplored as therapeutic targets for diabetes. This review comprehensively evaluates oGPCRs and their roles in diabetes. Orphan receptors are classified based on their functional groups: i) insulin secretagogues such as GPR119 and GPR142, ii) transcriptional and regulatory modulators such as GPR27, and iii) metabolic sensors such as GPR75 and GPR91. We further explored the biological roles of these receptors in the pancreas, adipose tissue, liver, and kidneys. In addition, we mapped the convergence of oGPCR signaling with the pharmacological properties of FDA-approved antidiabetic therapies. The major roles of incretin signaling, AMPK-mediated energy sensing, insulin secretion pathways, inflammatory signaling networks, and sodium-glucose cotransporter 2 (SGLT2) -associated organ protection are addressed. We also report the functionality of these receptors on a receptor-by-receptor basis and prioritize them based on biological relevance, druggability, and translational feasibility. Furthermore, potential repurposable drugs targeting oGPCR pathways, along with existing nondiabetic medications, are highlighted. Finally, we discuss how emerging technologies, including single-cell RNA sequencing, spatial transcriptomics, and artificial intelligence- and structure-based drug design, accelerate GPCR deorphanization and therapeutic development. Overall, this report highlights the significant role of oGPCRs as therapeutic targets and drug repurposing opportunities for developing precision medicine and emphasizes the need for further research on oGPCR signaling in the treatment of diabetes.

## 1 Introduction

Diabetes Mellitus (diabetes) is a chronic metabolic disease characterized by increased blood glucose levels due to decreased glucose regulation (1). This condition is caused by autoimmune cell destruction, leading to insulin deficiency or insulin resistance, where the body cannot respond to insulin signaling. The most common forms of diabetes are Type 1 (T1D) and Type 2 (T2D), which involve insulin deficiency and insulin resistance, respectively. T2D is the most common type of diabetes, accounting for 90% of all patients (2). Type 3c pancreatogenic diabetes is the third form of diabetes induced by exocrine pancreatic diseases, such as pancreatitis and pancreatic carcinoma (3). Other forms include gestational diabetes, which occurs during pregnancy, and genetic mutations that cause Maturity-Onset Diabetes of the Young (MODY) and Neonatal Diabetes (4).

The age at which diabetes affects individuals depends on the type of disease. Typically, T1D is diagnosed in children and adolescents, whereas T2D is commonly identified in middle and later life. T2D is particularly diagnosed in people over the age of 65 years and is closely linked to aging populations, obesity, and sedentary lifestyles (5). However, T2D also occurs in younger people, and it is estimated to increase by 178% by 2050 in children and adolescents in the United States alone (6). MODY is mostly diagnosed before the age of 25 years, while neonatal diabetes occurs before 6 months of age (7). It is estimated that approximately 537 million adults are affected by diabetes, which is fourfold higher than that four decades ago (8). Notably, early onset diabetes in childhood or adolescence often leads to more aggressive disease progression and is associated with a predicted loss of 15 years of life expectancy compared to nondiabetic individuals.

The constant increase in blood sugar levels and hyperglycemia can cause epigenetic changes, such as DNA methylation and histone modifications (9). These changes persist even after normal blood sugar levels are restored, leading to increased oxidative stress and inflammation. This chronic illness continues to increase due to the close interaction between diabetes and thyroid dysfunction (10). This combination can lead to metabolic instability and complex drug interactions. Youth-onset diabetes also tends to follow a more aggressive pathophysiological course than adult-onset diabetes. This metabolic mammary condition makes treatment more challenging in younger populations. Treatment failure is increasing across all age groups, while disease types and mortality continue to increase (11).

Therefore, there is an urgent need for new therapeutics that can cure the irreversible cellular damage caused by metabolic memory, in addition to decreasing glucose levels. One promising strategy for managing diabetes and associated endocrine comorbidities is targeting common molecular pathways, such as chronic inflammation, hormonal crosstalk, and mitochondrial failure. In addition, personalized, patient-specific approaches are needed to understand and prevent how diabetes can lead to cancer and brain-related diseases or treatment failure of these diseases (12).

This personalized medicine approach facilitates the understanding of the biological processes that link diabetes with complications such as carcinogenesis and cognitive dysfunction (12). The success of personalized medicine in diabetes depends on the molecular and signaling profiles of individual patients. Receptor-targeted therapies play a key role in this approach. The modulation of receptor signaling pathways potentially regulates glucose levels and controls the cellular metabolism. Receptor-specific treatments have been observed to improve therapeutic responses while reducing side effects (13). In general, among several receptor families, G protein-coupled receptors (GPCRs) are identified as important drug targets due to their central role in cellular signal transduction and physiological regulation. GPCR signaling regulates multiple metabolic pathways involved in insulin secretion, glucose homeostasis, and energy balance (14). Hence, modulating GPCR signaling can improve glycemic control while simultaneously influencing inflammation and metabolic dysfunction associated with diabetes. This necessitates the development of GPCR-targeted therapeutics to reduce disease progression and enhance the survival of patients with diabetes. Previously, it has been shown that several therapeutic agents, including antidiabetic drugs, can modulate GPCR signaling pathways that are clinically relevant (14). Orphan GPCRs (oGPCRs) have been also identified as biomarkers for many diseases, yet their functional roles are not fully characterized. These oGPCRs may regulate crucial pathways involved in diabetes pathophysiology (15). Thus, oGPCRs present an opportunity for the development of novel selective therapeutics for diabetes treatment.

This review provides a comprehensive analysis of oGPCRs and recently deorphanized GPCRs and their possible roles in diabetes. Specifically, the roles of oGPCRs in pancreatic β-cell function, insulin resistance, and metabolic regulation are discussed (15). The classification of oGPCRs, their biological functions, commonly shared pathways, and diabetes-associated oGPCRs were also examined. The therapeutic potential of oGPCRs, repurposable drugs targeting these receptors, clinical relevance, and feasibility have also been explored (16). By bridging molecular biology, pharmacology, and translational science, this review highlights the potential of oGPCRs as targets for developing diabetes therapeutics.

## 2 Search strategy

### 2.1 Literature search

A structured literature search was conducted in PubMed/MEDLINE, Scopus, Web of Science, Embase, and Google Scholar to identify relevant publications from January 2000 to July 2026. The search strategy combined Medical Subject Headings (MeSH) and free-text terms using Boolean operators (AND/OR). Keywords included “diabetes mellitus,” “type 1 diabetes,” “type 2 diabetes,” “gestational diabetes,” “GPCR,” “G protein-coupled receptor,” “orphan GPCR,” “adhesion GPCR,” “insulin secretion,” “insulin resistance,” “β-cell dysfunction,” “glucose homeostasis,” “drug repurposing,” “precision medicine,” “artificial intelligence,” “computational drug discovery,” “phytochemicals,” “plant-derived compounds,” and “natural products.” The reference lists of relevant articles were systematically examined to identify further eligible studies (17).

### 2.2 Study selection and eligibility criteria

Eligible publications included peer-reviewed original research articles, systematic reviews, meta-analyses, clinical studies, translational research, and seminal experimental investigations published in English between January 2000 and July 2026. Studies addressing GPCR signaling, diabetes pathogenesis, metabolic regulation, therapeutic target identification, drug discovery, plant-derived bioactive compounds, and emerging therapeutic approaches were included. The exclusion criteria comprised conference abstracts, editorials, letters to the editor, non-English publications, duplicate records, and studies that were not directly relevant to the review objectives. Retrieved records underwent an initial screening based on their titles and abstracts, followed by a full-text assessment to determine eligibility. Foundational studies published before 2000 were included if they provided critical mechanistic insights or established evidence relevant to the topics of this review.

### 2.3 Data extraction and evidence synthesis

Data were extracted using a standardized framework that aligned with the review objectives. The collected information included study type, experimental model or patient population, GPCR subtype, molecular targets, signaling pathways, metabolic outcomes and therapeutic implications. For studies evaluating approved or investigational therapeutics, additional variables included the drug name, original clinical indication, diabetes-related therapeutic status, primary molecular target (gene/protein), and associated signaling or metabolic pathways (s). For studies investigating natural products and phytochemicals, data extraction included plant-derived compounds (s), plant sources, target receptors (s), key signaling pathways (s), and metabolic and diabetes-related relevance. Where available, evidence of receptor modulation, glucose-lowering effects, insulin sensitivity, β cell function, anti-inflammatory actions, and cardiometabolic outcomes were also recorded. The extracted evidence was synthesized narratively to summarize the roles of GPCRs in diabetes pathophysiology, evaluate therapeutic and pharmacological opportunities, assess drug repurposing potential, and highlight the translational relevance of natural products, precision medicine strategies, and computational approaches for target identification and drug discovery.

## 3 Orphan GPCR biology, immune system and inflammation

oGPCRs are classified into three categories: Class A, Class B, and Adhesion class, which play unique roles in the pathophysiology of diabetes (14). Class A, the rhodopsin-like receptors, is the largest subfamily, which includes 83–87 non-olfactory members. Several Class A orphan receptors have been identified as significant metabolic regulators (14). For example, GPR119 is highly active in pancreatic β cells and intestinal L cells, which regulate glycemic conditions (18). Deletion of the orphan receptor GPR21 has been shown to improve insulin sensitivity and glucose tolerance in obese models (19). In addition, the GPR146 and GPR162 receptor pathways are involved in proinsulin C-peptide signaling and insulin exocytosis, respectively (20, 21). Similarly, GPR27 and GPR39 receptors are involved in insulin secretion and are downregulated in the adipose tissue of patients with obesity-related diabetes (22, 23).

Class B, the Secretin-like protein family, currently has no identified orphan members. However, this class is clinically important as a biomarker in diabetes, including glucagon, glucagon-like peptide-1 (GLP1), and GIP, which are primary targets for modern glucose-lowering therapies (13). The Adhesion GPCR (aGPCR) class, Class B2, consists of 33 receptors with a structurally conserved domain (24), the GPCR Autoproteolysis INducing domain (GAIN), and a built-in “Stachel” agonist sequence (25). These receptors play a crucial role in metabolic homeostasis. Specifically, ADGRF5 (GPR116) deficiency modulates insulin sensitivity and inhibits pancreatic islet development, whereas ADGRC2 (CELSR2) and ADGRC3 (CELSR3) are necessary for proper β-cell function (26). Additionally, ADGRL1 (LPHN1) receptors function as glucose receptors (27), and their loss is associated with increased food intake, obesity, and T2D-like features.

oGPCRs serve as important molecular links between metabolic dysfunction and the immune system, contributing to the chronic low-grade inflammation that characterizes both obesity and diabetes. Several oGPCRs, including GPR21, GPR84, and GPR35, play key roles in inflammation-driven insulin resistance (28). GPR84 functions as an immunemetabolic amplifier. Upon activation by medium-chain fatty acids, it promotes pro-inflammatory M1-like polarization of macrophages and neutrophils, leading to the release of cytokines such as tumor necrosis factor-alpha (TNF-α) and interleukin-1 beta (IL-1β). Similarly, constitutively active GPR21 promotes metabolic inflammation, and its genetic deletion has been shown to improve glucose tolerance and insulin sensitivity in obese animal models (19). Other oGPCRs regulate immune cell trafficking and tissue-specific inflammatory response. For example, GPR183 (EBI2) senses oxysterols to direct immune cell migration and modulate inflammatory signaling within the pancreatic islets (29). In contrast, GPR15 controls T-cell recruitment and homing to the mucosal tissues (30). In addition, the succinate receptor GPR91 (SUCNR1) promotes macrophage chemotaxis into visceral adipose tissue, thereby exacerbating obesity-associated inflammation. Conversely, the loss of the adhesion receptor GPR97 enhances macrophage inflammatory responses during obesity induced by a high-fat diet (31). Moreover, GPR50 deficiency is associated with increased adipose tissue inflammation and impaired insulin signaling (32). Together, these findings highlight the central role of oGPCRs in integrating immune regulation with metabolic homeostasis and identify them as promising therapeutic targets for diabetes and other metabolic inflammatory disorders.

In T1D, receptors such as GPR65 (TDAG8) and GPR183 (EBI2) are important regulators of immune responses (33, 34). GPR183 functions as an oxysterol sensor that directs immune cell migration and regulates inflammatory signaling in pancreatic islets. In addition, oGPCRs are emerging as critical molecular mediators in both autoimmune destruction associated with T1D and chronic low-grade inflammation that drives T2D. GPR65 is genetically associated with T1D and modulates autoimmune activity through invariant natural killer T (iNKT) cells (35). In addition, single-cell RNA sequencing studies have identified oGPCRs, including GPR119 (36), and genetic variants of MTNR1B as valuable biomarkers (37). These markers help identify rare cell populations involved in autoimmune responses and may improve the prediction of disease progression in patients with ITP. In T2D, several oGPCRs, including GPR21, GPR84, and GPR35, serve as important molecular links between adipose tissue expansion, chronic inflammation, and insulin resistance. GPR21 is a constitutively active receptor that promotes metabolic and inflammatory responses. Genetic deletion of GPR21 has been shown to improve glucose tolerance and insulin sensitivity in experimental animal models. Similarly, GPR84 functions as an immune metabolic amplifier. Activation of GPR84 by medium-chain fatty acids promotes pro-inflammatory M1-like polarization of macrophages (38). It also stimulates the release of inflammatory cytokines, including tumor necrosis factor-alpha (TNF-α and IL-1β. Furthermore, the succinate receptor GPR91 promotes macrophage chemotaxis in the visceral adipose tissue, thereby exacerbating obesity-associated inflammation (39). In contrast, the loss of the adhesion receptor GPR97 enhances macrophage inflammatory responses during obesity induced by a high-fat diet. GPR50 deficiency is associated with increased adipose tissue inflammation and impaired insulin signaling. Collectively, these findings demonstrate that oGPCRs are key regulators of immune and metabolic pathways in diabetes (32). They also highlight the potential of these receptors as promising therapeutic targets for next-generation immunomodulatory and anti-inflammatory therapies for T1D and T2D. In this review, we highlight the roles of oGPCRs in insulin resistance and obesity, discuss the emerging functions of adhesion GPCRs (aGPCRs) and other orphan receptors implicated in diabetes, and examine the broader oGPCR landscape and its convergence with key metabolic pathways involved in diabetes.

## 4 Orphan GPCRs regulating pancreatic β-cell function

**Figure 1:**
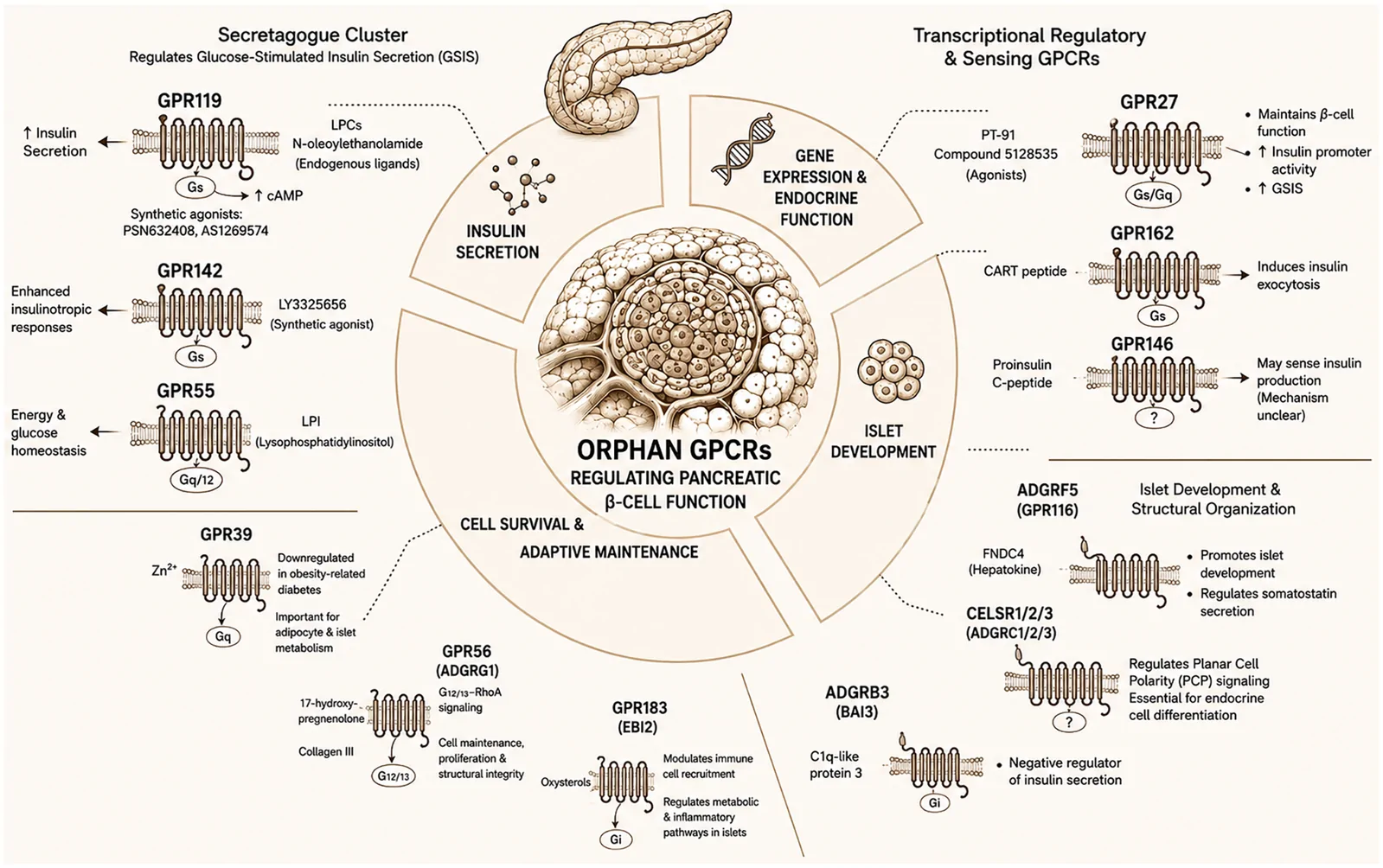
Orphan GPCRs in pancreatic β−cell regulation: Orphan GPCRs form an integrated network controlling pancreatic β−cell function across insulin secretion, gene expression, islet development, and cell survival. Secretagogue receptors (GPR119, GPR142, GPR55, and GPR39) enhance GSIS and metabolic responses, whereas regulatory/sensing GPCRs (GPR27, GPR162, and GPR146) modulate insulin expression and exocytosis. Adhesion and developmental GPCRs (ADGRF5, CELSR1/2/3, and ADGRB3) govern islet organization and differentiation, and survival-associated receptors (GPR56, GPR183, and GPR39) maintain β-cell integrity and adaptive responses. Collectively, these pathways highlight orphan GPCRs as key modulators and therapeutic targets for diabetes.

In diabetes, pancreatic islet dysfunction is characterized by reduced insulin production from β-cells and abnormal glucagon release from α cells (14). In T1D, autoimmune-mediated destruction of pancreatic β-cells causes insulin deficiency, whereas in T2D, insulin resistance develops first, followed by β-cell dysfunction, leading to insulin deficiency (14). Consequently, disruption of glucose homeostasis leads to chronic hyperglycemia and induces metabolic and vascular diseases. Molecular characterization has shown that numerous oGPCRs are expressed in pancreatic cells, where they play key roles in regulating metabolic processes (14). oGPCRs can be classified into specific groups based on their functions, such as the regulation of insulin secretion, endocrine cell growth, and preservation of islet structure (**Figure 1**).

Briefly, the secretagogue cluster, comprising receptors such as GPR119, GPR142, and GPR55, is involved in glucose-stimulated insulin secretion (GSIS) (40). The recently deorphanized GPR119 is a lipid-sensing receptor that activates Gs/cAMP signaling upon binding to endogenous ligands, such as lysophosphatidylcholines (LPCs) and N-oleoylethanolamide (36). Its pharmacological relevance in glycemic regulation is also supported by the activity of synthetic agonists, including PSN632408 and AS1269574 (41). Similarly, human clinical studies have shown that GPR142 interaction with the synthetic agonist LY3325656 leads to enhanced insulinotropic responses (42). Another orphan receptor, GPR55, functions as a non-canonical metabolic regulator that responds to lysophosphatidylinositol (LPI), thereby contributing to the maintenance of energy and glucose homeostasis (43).

The regulation of islet gene expression and endocrine function involves a second group of receptors, which includes transcriptional regulatory and sensing GPCRs. The GPR27 receptor has been shown to maintains β-cell functionality. GPR27 knockdown in murine models showed a decrease in insulin promoter activity and altered GSIS. Recent reports provide evidence for the use of the agonists PT-91 and compound 5128535 to regulate GPR27 function (44). In contrast, GPR162 has been proposed as a target receptor for the cocaine- and amphetamine-regulated transcript (CART) peptide, which induces insulin exocytosis in pancreatic cells (45). GPR146 may sense insulin production, although the mechanisms of signal transduction remain unclear (46).

Another notable receptor family is the adhesion GPCR (aGPCR), which also plays a significant role in islet development and structural organization. These receptors function through unique activation mechanisms, such as the “Stachel” agonist sequence. The ADGRF5 (GPR116) receptor is activated by the hepatokine FNDC4, and its deficiency in animal models reduces islet development and dysregulates somatostatin secretion (47). Members of the cadherin, epidermal growth factor (EGF) LAG seven-pass G-type receptor (CELSR) include CELSR1 CELSR2 and CELSR3 (48). This subfamily regulates planar cell polarity (PCP) signaling, which is essential for endocrine cell differentiation during pancreatic development. In contrast, ADGRB3 (BAI3) has been shown to functions as a negative regulator of insulin secretion upon activation by its ligand, C1q-like protein 3 (49).

Finally, a set of receptors contributes to cellular survival and adaptive maintenance in the endocrine pancreas. GPR39 Deregulation of The GPR39 receptor in animal models leads to adverse effects on adipocyte metabolism (50). GPR183, also known as EBI2, regulates immune cell recruitment through oxysterol sensing and modulates metabolic and inflammatory pathways in the islets (29). The receptor GPR56 (ADGRG1) also plays a crucial role in diabetes. It is a steroid-responsive receptor activated by ligands such as 17-hydroxypregnenolone and collagen III (51). It is regulated by the G-protein subtype G_12_/_13_-RhoA signaling pathway, which is involved in cellular maintenance, proliferation, and structural integrity. In addition, selective small-molecule modulators, such as AP503 and GL64, are being explored to target adhesion GPCRs for therapeutic applications in metabolic and skeletal diseases (25).

## 5 Orphan GPCRs involved in insulin resistance and obesity

### 5.1 Inflammation-driven insulin resistance: roles of GPR21, GPR84, and GPR35

**Figure 2:**
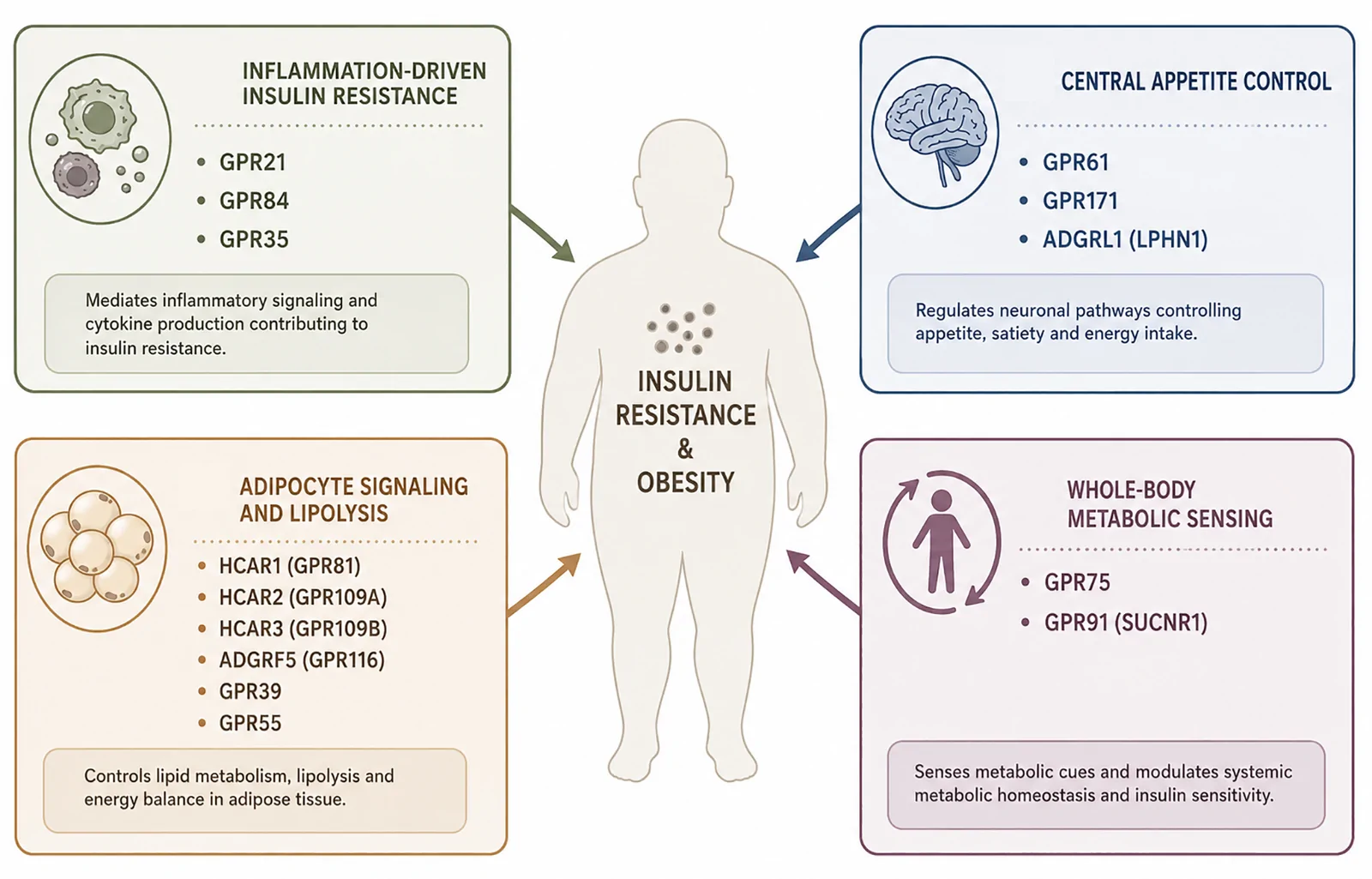
Orphan GPCRs in insulin resistance and obesity: Orphan GPCRs contribute to insulin resistance and obesity through interconnected pathways involving inflammation, central appetite control, adipocyte signaling, and systemic metabolic sensing. Inflammatory GPCRs (GPR21, GPR84, and GPR35) promote insulin resistance, whereas central receptors (GPR61, GPR171, and ADGRL1) regulate appetite and energy balance. Adipocyte-associated GPCRs (HCAR1/2/3, ADGRF5, GPR39, GPR55) control lipolysis and fat metabolism, and systemic sensors (GPR75, GPR91) integrate whole−body metabolic responses. Together, these receptor classes form a coordinated network that drives metabolic dysfunction and represents a potential therapeutic target.

Chronic low-grade inflammation is a primary driver of systemic insulin resistance (IR) in obesity (52). It is often induced by the expansion of visceral adipose tissue, which triggers the recruitment of immune cells and the secretion of pro-inflammatory cytokines, such as TNF-α, IL-1β, and IL-6 (53). oGPCRs, such as GPR21, GPR84, and GPR35, serve as vital links between chronic low-grade inflammation and the development of insulin resistance (IR) in obesity (28) (**Figure 2**). GPR21 is a constitutively active Class A receptor that signals through Gα 15/16 and phospholipase C (PLC) to activate the mitogen-activated protein kinase (MAPK) cascade. GPR21 is overexpressed in adipose tissue during high-fat and high-sugar feeding. Its downstream signaling regulates the c-Jun N-terminal kinase (JNK) pathway (19). Consequently, insulin resistance leads to phosphorylation of insulin receptor substrate 1 (IRS1) at Ser307, thereby reducing insulin-stimulated tyrosine phosphorylation and downstream glucose uptake (54). Structural analysis has also identified an agonist-like motif in extracellular loop 2 (ECL2) that maintains high basal activity and insulin sensitivity (55).

Another type of receptor, GPR84, is involved in pro-inflammatory processes triggered by medium-chain fatty acids (MCFAs) (56). This receptor is overexpressed in macrophages and neutrophils and regulates the Gαi/o and PLC/Ca^2+^ pathways. This promotes M1-like pro-inflammatory polarization and the secretion of cytokines, such as TNF-α and IL-1β (57). In adipose tissue, activation of GPR84 by its agonist decanoic acid (capric acid, C10:0) inhibits the secretion of adiponectin, an insulin-sensitizing hormone, thereby worsening systemic insulin resistance (58). GPR35 is also linked to T2D through various single-nucleotide polymorphisms and contributes to metabolic homeostasis via Gα12/13 and Gαi/o signaling. GPR35 agonists, such as kynurenic acid and lysophosphatidic acid (LPA), regulate glucose uptake and intestinal immune responses, which improves oral glucose tolerance in diabetic models (59). Overall, these receptors form a gene regulatory network that integrates fatty acid sensing and immune signaling in the pathophysiology of obesity-associated metabolic dysfunction.

### 5.2 Central appetite control: roles of GPR61, GPR171, and ADGRL1

Central appetite and energy homeostasis are also regulated by oGPCRs signaling in the hypothalamus. Several receptors are linked to nutrient and hormonal signals that directly contribute to obesity and systemic IR. The orphan receptor GPR61 is a Gs-coupled receptor expressed in the hypothalamic regions, including the arcuate (ARC) and ventromedial (VMH) nuclei (60) (**Figure 2**). Deletion of the GPR61 protein-coding gene in mice leads to hyperphagia and obesity (61). These animals show a significant decrease in appetite-suppressing molecules, such as proopiomelanocortin (POMC) and brain-derived neurotrophic factor (BDNF) (62). In humans, GPR61 function is associated with increased body mass index (BMI) and body weight (63). Severely obese individuals often carry loss-of-function missense mutations of R236C5.66 in GPR61, which inhibits cAMP production (63). The receptor GPR171 also functions as a hypothalamic receptor for the proSAAS-derived neuropeptide BigLEN (64). It is a Gi/o-coupled receptor that inhibits adenylyl cyclase, leading to the regulation of feeding behavior. Knockdown of GPR171 in the ventral hypothalamus alters metabolic balance. Specifically, it increases the respiratory exchange ratio (RER), heat production, and food intake (64). Furthermore, the adhesion GPCR ADGRL1 activates Gαi signaling and functions as a glucose receptor (65). It is mainly expressed in ventromedial hypothalamus (VMH) neurons, and its aberration affects glucose sensing. This leads to vagally mediated fasting hyperinsulinemia, chronic insulin resistance, and obesity. Overall, these receptors appear to play a crucial role in metabolic imbalance and contribute to the development of T2D.

### 5.3 Adipocyte signaling and lipolysis: roles of HCARs, GPR39, and GPR55

oGPCRs have also been identified as significant regulators of adipocyte signaling and lipolysis. These receptors are involved in metabolic signaling to maintain energy homeostasis. Specifically, the hydroxy-carboxylic acid receptors HCAR1 (GPR81), HCAR2 (GPR109A), and HCAR3 (GPR109B) play important roles in reducing fat breakdown in adipose tissue (66) (**Figure 2**). These Gαi proteins inhibit cAMP production, followed by the inhibition of triglyceride lipase activity. These receptors respond to specific metabolic intermediates, and HCAR1 is activated by lactate, a glycolysis product (66). This creates an autocrine feedback loop in which insulin-stimulated glucose uptake increases lactate production, which inhibits further lipolysis. In contrast, HCAR2 is activated by the ketone body β-hydroxybutyrate (BHB). It acts as a negative feedback regulator that preserves energy during fasting. Obese individuals have been observed to have lower HCAR2 expression in subcutaneous adipose tissue. Low expression is also associated with lipid mobilization and metabolic syndrome (67).

Beyond the HCA family, the receptors GPR39 and GPR55 also play functionally important roles in adipocyte activity and systemic insulin sensitivity (**Figure 2**). Its downstream signaling is activated through Gαq, Gα12/13 pathways, and Gαs signaling upon the binding of zinc ions. Studies have shown that GPR39 expression is downregulated in the adipose tissue of individuals with obesity-related diabetes (50). In mouse models, genetic disruption of GPR39 leads to increased body fat and altered adipocyte metabolism (50). In contrast, GPR55 is an atypical lipid receptor activated by L-α-lysophosphatidylinositol (LPI). It is often upregulated in the adipose tissue of obese and diabetic patients. GPR55 signals through Gαq/11 and Gα12/13 pathways. While it is linked to hepatic steatosis, its role in adipose tissue involves the regulation of energy expenditure. Notably, global deletion of GPR55 promotes obesity by reducing physical activity (43).

Furthermore, the adhesion GPCR ADGRF5 (GPR116) plays a key role in the liver-white adipose tissue endocrine axis (**Figure 2**) (68). Activation of this receptor in white adipocytes triggers the Gαs-cAMP signaling pathway. This enhances insulin-mediated glucose uptake and improves the systemic glucose tolerance. This signaling axis is significantly reduced in individuals with prediabetes and diabetes. However, GPR116 expression in subcutaneous adipose tissue can recover after weight loss following bariatric surgery. In addition to GPR116, GPR39 is involved in improving the body’s response to insulin and reducing metabolic problems linked to obesity. Thus, targeting these pathways may offer promising therapeutic strategies (69). For example, the interaction of MK-0354 with GPR109A (70) and FNDC4 with GPR116 provides opportunities to treat insulin resistance and obesity without the side effects of conventional therapies (68).

### 5.4 Whole-body metabolic sensing: roles of GPR75 and GPR91

Whole-body metabolic sensors, including oGPCRs GPR75 and GPR91, act as major regulators that translate metabolite changes into physiological responses (40) (**Figure 2**). These receptors play a significant role in obesity and IR progression. Large-scale human exome sequencing has predicted loss-of-function (pLOF) variants of GPR75, which are strongly associated with protection against obesity (71). Individuals carrying these mutations have a lower body mass index (BMI) and up to a 54% reduced risk of obesity (71). Similar features have also been observed in animal models. Gpr75 knockout mice fed a high-fat diet (HFD) exhibited a lean phenotype, reduced adiposity, and significantly improved insulin sensitivity and glucose tolerance. GPR75 expression was found in peripheral tissues such as vascular endothelium and adipose tissue. It is also overexpressed in the hypothalamus and localizes to primary cilia in neurons, which are essential for sensing energy-related signals in the body. At the molecular level, the ligand 20-hydroxyeicosatetraenoic acid (20-HETE) has a high affinity for GPR75 and activates the Gαq/11 signaling cascade (72). This leads to the activation of phospholipase C, increased intracellular calcium levels, and protein kinase C (PKC) activation. This signaling pathway contributes to insulin resistance in patients with metabolic syndrome. Additionally, GPR75 activation increases vascular angiotensin-converting enzyme (ACE) expression and promotes oxidative stress. This shows that obesity-related hypertension is part of a broader metabolic dysfunction. In contrast, the chemokine CCL5 (Regulated upon Activation, Normal T cell Expressed and Secreted (RANTES)) may act as a low-affinity inhibitor of GPR75 (73). This potentially functions as a counteracting mechanism against excessive 20-HETE signaling.

In parallel, GPR91 functions as a sensor for succinate, which is regulated by the tricarboxylic acid (TCA) cycle (40). Succinate accumulates in the circulation and tissues under hypoxic, metabolic stress, and hyperglycemic conditions. Succinate ligands bind to GPR91 and inhibit lipolysis through Gαi signaling, thereby regulating free fatty acid (FFAs) release (74). While this mechanism contributes to metabolic homeostasis under normal conditions, it is often dysregulated in obese individuals. Patients with metabolic syndrome also have higher plasma succinate levels (74). Moreover, GPR91 deficiency in HFD-fed mice leads to hyperglycemia and reduced glucose clearance (74). Beyond metabolic regulation, GPR91 is involved in immune and inflammatory processes. Succinate released from hypoxic adipocytes induces macrophage recruitment into the visceral adipose tissue via the GPR91-mediated chemotaxis mechanism. Elevated succinate levels have also been observed in diabetic nephropathy. In non-alcoholic fatty liver disease (NAFLD), this pathway induces the activation of hepatic stellate cells and fibrosis (39). GPR91 signaling also contributes to multi-organ complications associated with obesity and systemic insulin resistance (75). Overall, GPR75 and GPR91 appear to share common pathways and functions in metabolic diseases (**Figure 2**).

## 6 Adhesion GPCRs & emerging orphan receptors in diabetes

The second-largest subfamily of GPCRs, aGPCRs have emerged as key regulators of metabolic and endocrine homeostasis (76). Structurally, they are characterized by large N-terminal extracellular regions (ECRs), which extend to thousands of amino acids. These long-chain amino acids contain diverse adhesive components, including lectin, laminin, and cadherin domains, which are involved in cell-cell and cell-matrix interactions. The GAIN domain of aGPCRs promotes self-catalyzed cleavage at the GPCR proteolysis site (GPS) (76). Subsequently, it produces non-covalently linked N-terminal- and C-terminal fragments, which is a unique structural characteristic. These receptors function as mechanosensors that regulate the signal transduction pathway through the internal agonistic “Stachel” sequence (76). The detailed functional roles of aGPCRs in metabolic tissues are summarized below.

### 6.1 Adhesion GPCRs

GPR56 is the most abundantly expressed GPCR mRNA in human pancreatic islets and increases glucose-stimulated insulin release (77). It also enhances β-cell survival and proliferation through the cAMP/PKA signaling pathway. GPR97 (ADGRG3) plays a role in immune–metabolic interactions and is elevated during systemic inflammation; its absence enhances macrophage inflammatory responses in obesity induced by a high-fat diet (31). GPR126 (ADGRG6) links tissue remodeling with metabolic regulation and is crucial for developmental processes, such as Schwann cell myelination and bone mineralization, in addition to being expressed in the liver (78). ADGRF1 (GPR110) regulates hepatic lipid metabolism and is predominantly expressed in hepatocytes, where it regulates SCD1 expression. Although it is downregulated in obesity, its overexpression aggravates hepatic steatosis and non-alcoholic fatty liver disease (NAFLD) by amplifying *de novo* lipogenesis (79). ADGRF5 (GPR116) is involved in the liver–adipose endocrine axis, functioning as a receptor for FNDC4 in white adipose tissue, thereby improving insulin sensitivity and glucose uptake (68). ADGRA1 (GPR123) is specifically expressed in the hypothalamus and exerts a detrimental influence on energy expenditure and thermogenesis through the sympathetic nervous system and thyroid axis (49). ADGRL1 functions as a hypothalamic glucose sensor within the ventromedial nucleus and is essential for the regulation of insulin levels (80). Its absence causes hyperphagia, obesity, and insulin resistance due to compromised glucose sensing. These orphan receptors exhibit various sensory and regulatory functions in metabolic physiology and serve as promising therapeutic targets for metabolic syndrome and type 2 diabetes.

### 6.2 Emerging orphan GPCRs in diabetes

Beyond the family of aGPCRs, several oGPCRs have emerged as crucial receptors in diabetes, involved in nutrient sensing and endocrine signaling. GPR146 is an emerging oGPCRs that colocalizes with C-peptide at the cell membrane and regulates signaling pathways involved in microvascular function (20, 21). It also regulates the side effects of diabetes, such as retinopathy. In contrast, the overexpressed GPR142 protein regulates nutrient-driven insulin secretion in pancreatic islets (81). This receptor senses aromatic amino acids L-tryptophan and enhances glucose-dependent insulin secretion through Gαq-mediated signaling. This signaling pathway leads to insulin release with a reduced risk of hypoglycemia. In contrast, GPR84 functions as an immune metabolic amplifier (82). It is activated by medium-chain fatty acids and is upregulated in obesity and non-alcoholic fatty liver disease, where it promotes immune cell infiltration and contributes to pathological fibrosis in metabolic tissues, such as the liver and kidney. The succinate-GPR91 axis enhances insulin secretion in a glucose-dependent manner in β-cells, and its increased expression in obese and diabetic islets highlights its role in metabolic dysregulation.

Central and peripheral metabolic regulation also involves GPR158 and GPR160 (83, 84). GPR160 participates in anorexigenic signaling mediated by cocaine- and amphetamine-regulated transcript in the brain, while related signaling pathways contribute to β-cell and neuroendocrine control of energy balance (83). Functioning as a metabolic hub, GPR39 modulates insulin secretion and β-cell survival, and its dysregulation is associated with impaired energy expenditure, obesity, and insulin resistance (22, 23). Similarly, the HCAR1–3 family regulates adipose tissue lipolysis by sensing metabolites such as β-hydroxybutyrate and lactate, thereby regulating fat storage and mobilization (85). Overall, oGPCRs play a significant role in nutrient, metabolite, and hormonal signaling, providing an opportunity to target these receptors for developing next-generation diabetes therapeutics.

## 7 Orphan GPCR landscape and its convergence with core metabolic pathways in diabetes

FDA-approved antidiabetic drugs act through six major physiological pathways. These pathways potentially regulate glucose homeostasis in the pancreatic, hepatic, renal, and enteroendocrine systems. As described above, one of the significant mechanisms involves the stimulation of insulin secretion from pancreatic β cells. This pathway is targeted by KATP channel modulators, including sulfonylureas (e.g., glipizide and glyburide) and meglitinides (e.g., repaglinide). These modulators inhibit the KATP channel subunits KCNJ11 and ABCC8, leading to membrane depolarization of β-cells (86). Depolarization regulates Ca^2+^ influx and subsequent insulin exocytosis. This mechanism enhances endogenous insulin release and improves glycemic control in T2D patients. Incretin GPCR signaling is amplified by GLP1 receptor (GLP1R) agonists (liraglutide, semaglutide, exenatide) (87) and dipeptidyl peptidase-4 (DPP-4) inhibitors (sitagliptin, linagliptin) (88), which enhance GLP1R-mediated cAMP-PKA signaling to increase glucose-dependent insulin secretion and suppress glucagon release. Several antidiabetic drugs reduce blood glucose levels by targeting hepatic glucose production, peripheral insulin sensitivity, renal glucose reabsorption, and enteroendocrine signaling pathways. Briefly, Metformin reduces hepatic glucose production by inhibiting mitochondrial complex I, which suppresses the gluconeogenic genes PEPCK and G6Pase (89). Pioglitazone and rosiglitazone improve insulin sensitivity through PPARγ activation, thereby enhancing glucose uptake in peripheral tissues (90). Empagliflozin, dapagliflozin, and canagliflozin lower blood glucose levels by inhibiting SGLT2 (SLC5A2) in the proximal tubule (91). It further reduces renal glucose reabsorption and promotes insulin-independent glycosuria. Finally, colesevelam modulates bile acid-GPCR signaling via TGR5-linked pathways. This leads to increased GLP1 secretion and improved postprandial glucose control (92). Overall, targeting these core pathways may provide a modern diabetes therapy to control and treat diabetes.

**Figure 3:**
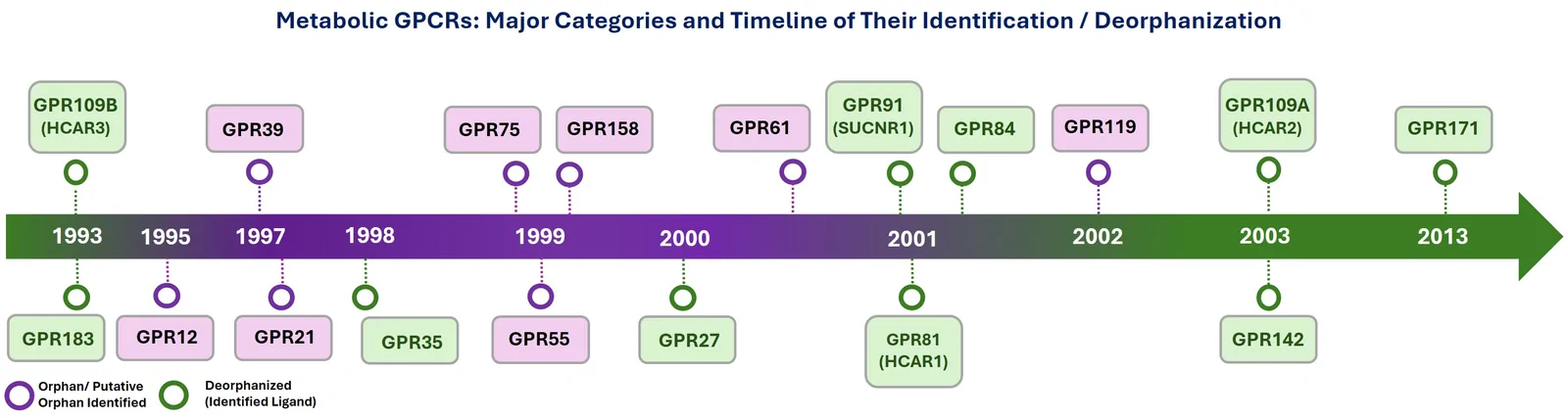
Timeline of identification and deorphanization of metabolic G protein-coupled receptors (GPCRs). Metabolic GPCRs are shown according to the year of their initial identification as orphan receptors and/or subsequent deorphanization. Purple symbols and boxes indicate orphan or putative orphan GPCRs at the time of discovery, whereas green symbols and boxes denote receptors for which endogenous ligands have been identified (deorphanized receptors). The receptors can be broadly categorized into two major functional clusters: Cluster 1, comprising peripheral metabolic and endocrine GPCRs involved in insulin secretion, incretin signaling, lipid sensing, and metabolic inflammation (GPR39, GPR55, GPR119, GPR142, GPR35, GPR81/HCAR1, GPR109A/HCAR2, GPR109B/HCAR3, GPR84, and GPR91/SUCNR1); and Cluster 2, consisting of central neuro-metabolic GPCRs associated with appetite regulation, energy expenditure, and neuroendocrine integration (GPR12, GPR21, GPR27, GPR61, GPR75, GPR158, and GPR171). An additional immune-inflammatory cluster, represented by receptors such as GPR183 and GPR84, is linked to macrophage recruitment, cytokine production, and inflammatory signaling pathways associated with metabolic dysfunction. The timeline illustrates the progression of receptor discovery and functional characterization from 1993 to 2013.

Briefly, the first cluster of oGPCRs (**Figure 3**), including class A metabolic receptors, lipid-sensing GPCRs, and adhesion GPCRs, commonly shares many physiological pathways targeted by antidiabetic therapies (14). These pathways include incretin signaling, insulin secretion, energy sensing and immunometabolic regulation. Several receptors, such as GPR119, GPR142, GPR55, GPR39, and GPR35, converge on β-cell insulin secretion and enteroendocrine incretin pathways (14). In particular, GPR119 and GPR142 enhance glucose-stimulated insulin release via Gs-cAMP-PKA signaling, functionally overlapping with the GLP1R/DPP-4 incretin axis. Lipid- and metabolite-sensing receptors, including HCAR1, HCAR2, HCAR3, and GPR84, are closely associated with metabolic inflammation and adipose insulin sensitivity (56). These receptors connect short-chain fatty acids and lipid intermediates to AMPK activation, NF-κB suppression, and insulin resistance. Their signaling mechanisms also overlap with the metabolic effects of metformin and PPARγ-targeting drugs. Similarly, GPR91 links succinate accumulation to renal and retinal complications via Gq-mediated inflammatory and hypoxic signaling (40). In addition, it functionally overlaps with the protective effects of SGLT2 inhibitors in diabetic kidney disease (93).

The second functional cluster of oGPCRs (**Figure 3**) mainly regulates central appetite control, energy expenditure, and neuro-metabolic integration (94). This cluster includes neuroendocrine and hypothalamic GPCRs, such as GPR12, GPR21, GPR27, GPR61, GPR75, GPR158, and GPR171 (94). This group of drugs functionally features in a similar way to incretin-based treatments and dopamine-related metabolic regulation, including bromocriptine. In addition to these clusters, immune- and inflammation-associated GPCRs, such as GPR183 and GPR84, form another distinct cluster (95). These receptors participate in macrophage recruitment and cytokine production. They directly intersect with the NF-κB, IL-1, and TNF inflammatory pathways (95). These pathways may also serve as therapeutic targets for repurposed anti-inflammatory drugs in diabetes.

The adhesion GPCR family includes ADGRF5 (GPR116), ADGRB3 (BAI3), ADGRC2 (CELSR2), ADGRC3 (CELSR3), GPR56 (ADGRG1), GPR97 (ADGRG3), GPR126 (ADGRG6), ADGRF1 (GPR110), and ADGRA1. This family primarily contributes to vascular integrity, endothelial signaling, immune cell adhesion and tissue remodeling. These functions align with the pathways modulated by RAAS inhibitors, such as AGTR1 blockade (96), as well as PPAR agonists and SGLT2-mediated organ protection (97). These receptors also converge on the PI3K-AKT survival signaling, endothelial barrier regulation, and inflammatory cell trafficking pathways. These processes are central to the progression of diabetic nephropathy, retinopathy, and cardiomyopathy.

Overall, these GPCR networks integrate with the major therapeutic pathways targeted by FDA-approved antidiabetic drugs, including incretin GPCR signaling through the GLP1 axis with agents such as semaglutide and liraglutide, AMPK energy sensing with metformin, insulin secretion circuits with sulfonylureas such as glimepiride, lipid inflammatory coupling involving NF-kappa B and NLRP3 modulated by agents such as pioglitazone and anakinra (98), and renal vascular protective signaling through SGLT2 inhibitors such as empagliflozin (99) and RAAS modulators such as losartan (100). A more detailed status of currently approved drugs and their mechanistic classification is discussed below.

## 8 Current antidiabetic drugs, mechanisms and clinical applications

Antidiabetic drugs comprise a diverse group of pharmacological agents used to manage hyperglycemia in diabetes mellitus (101). They include insulin preparations and multiple classes of oral and injectable therapies, such as metformin; sulfonylureas (e.g., glimepiride, glipizide); meglitinides (repaglinide, nateglinide); thiazolidinediones (pioglitazone, rosiglitazone); GLP1R agonists (exenatide, liraglutide, dulaglutide, semaglutide, lixisenatide); dual incretin agonist tirzepatide; DPP-4 inhibitors (sitagliptin, saxagliptin, linagliptin, alogliptin, vildagliptin); SGLT2 inhibitors (empagliflozin, dapagliflozin, canagliflozin, ertugliflozin); alpha-glucosidase inhibitors (acarbose, miglitol, voglibose); amylin analog pramlintide; and additional agents such as bromocriptine and colesevelam (101). These drugs exert their effects through distinct yet complementary mechanisms, including enhancement of insulin secretion, improvement of insulin sensitivity, modulation of incretin signaling, delay of intestinal carbohydrate absorption, suppression of hepatic gluconeogenesis, and promotion of urinary glucose excretion, thereby achieving glycemic control and reducing the risk of diabetes-related complications. Here, we explored the drugs currently employed for the treatment of diabetes. In addition, other potential candidates that may be repurposed for its management are reviewed.

GLP1R agonists, DPP-4 inhibitors, and SGLT2 inhibitors are key antidiabetic drug classes that regulate glucose levels through complementary mechanisms (101). GLP1R agonists induce insulin secretion, suppress glucagon levels, and reduce appetite. DPP-4 inhibitors increase endogenous GLP1 activity by preventing its degradation and improving postprandial glycemic control (13). SGLT2 inhibitors reduce blood glucose levels by promoting renal glucose excretion independently of insulin (101). Together, these provide effective glycemic control with additional cardiovascular and renal benefits in T2D management.

GLP1R agonists, including liraglutide, semaglutide, and exenatide, have been identified as potential therapies for T2D. GLP1-ligand interaction enhances glucose-dependent insulin secretion, suppresses glucagon release, and preserves pancreatic β-cell function. Beyond glycemic control, these drugs have been successfully repurposed for obesity management owing to their appetite-suppressing effects. GLP1-ligand interactions provide significant cardiovascular and renal protection (13). Specifically, it reduces adverse cardiovascular events and slows the progression of chronic kidney disease. Experimental evidence also reports that GLP1-ligand activation facilitates the treatment of neurodegenerative disorders, including Parkinson’s and Alzheimer’s disease. Furthermore, DPP-4 inhibitors, such as sitagliptin and linagliptin, increase glycemic control by preventing the degradation of endogenous incretin hormones (102). DPP-4 inhibitors are being investigated for their antiviral and anticancer potential. However, combining DPP-4 inhibitors with GLP1R agonists is generally not recommended, as this combination provides minimal additional glycemic benefit (103). Similarly, SGLT2 inhibitors, such as canagliflozin and empagliflozin, potentially reduce blood glucose levels by inhibiting renal glucose reabsorption. These drugs have evolved beyond glucose-lowering therapies to include the treatment of heart failure and chronic kidney disease. SGLT2 inhibitors also modulate lipid profiles by reducing triglyceride levels and increasing the levels of high-density lipoprotein cholesterol. Notably, the combinatorial effect of GLP1 agonists with SGLT2 inhibitors provides an alternative therapy for treating cardiovascular and renal diseases, thus offering better opportunities for metabolic disease management (104). Overall, the combination therapy of these drugs, along nondiabeticon-diabetic drugs, may offer a more effective approach for metabolic disease management (**Table 1**).

**Table 1:** Summary of antidiabetic drugs.

Drug	Original indication	Diabetes status	Primary target (gene/protein)	Major pathway	Key reference
Metformin	Antihyperglycemic (historic plant-derived origin)	Approved (T2D)	PRKAA1/2 (I	AMPK–mTOR, hepatic gluconeogenesis ↓	(105)
Bromocriptine	Parkinson disease, hyperprolactinemia	Approved (T2D)	DRD2	Dopamine–hypothalamic circadian control	(106)
Colesevelam	Hyperlipidemia	Approved (T2D adjunct)	FXR/TGR5	Bile acid–GLP1 axis	(107)
Hydroxychloroquine	Malaria, lupus, RA	Approved (India)/off-label	TLR7, TLR9	NF-κB suppression	(108)
Verapamil	Hypertension, arrhythmia	Phase II/III (T1D/T2D)	CACNA1C	TXNIP inhibition, β-cell preservation	(109)
Sildenafil	Erectile dysfunction	Investigational	PDE5A	cGMP–PKG signaling	(110)
Tadalafil	Erectile dysfunction	Investigational	PDE5A	cGMP pathway	(111)
Allopurinol	Gout	Investigational	XDH/XO	Oxidative stress reduction	(112)
Salsalate	Anti-inflammatory analgesic	Phase III trials	IKBKB	NF-κB inhibition	(113)
Colchicine	Gout	Investigational	Tubulin, NLRP3	Inflammasome inhibition	(114)
Anakinra	Rheumatoid arthritis	Clinical studies	IL1R1	IL-1 signaling blockade	(115)
Canakinumab	Auto-inflammatory disease	Investigational	IL1B	IL-1β inhibition	(116)
Tocilizumab	Rheumatoid arthritis	Investigational	IL6R	JAK–STAT pathway	(117)
Etanercept	Rheumatoid arthritis	Experimental	TNF	TNF signaling inhibition	(118)
Infliximab	Crohn disease, RA	Experimental	TNF	TNF signaling	(119)
Adalimumab	Rheumatoid arthritis	Experimental	TNF	TNF pathway	(120)
Rituximab	Lymphoma, RA	Phase II (T1D)	CD20 (MS4A1)	B-cell depletion	(121)
Abatacept	Rheumatoid arthritis	Phase II (T1D)	CD80/CD86	T-cell co-stimulation blockade	(122)
Teplizumab	Autoimmune therapy	FDA-approved (T1D delay)	CD3E	T-cell modulation	(123)
Imatinib	CML	Investigational	ABL1, KIT, PDGFRA	ER stress reduction	(124)
Telmisartan	Hypertension	Investigational	AGTR1, PPARG	RAAS + insulin sensitization	(125)
Losartan	Hypertension	Adjunct	AGTR1	RAAS inhibition	(126)
Ramipril	Hypertension	Prediabetes studies	ACE	RAAS blockade	(127)
Fenofibrate	Hypertriglyceridemia	Adjunct (retinopathy)	PPARA	Lipid oxidation	(128)
Bezafibrate	Dyslipidemia	Investigational	PPARA/PPARG/PPARD	Lipid-glucose metabolism	(129)
Naltrexone	Opioid dependence	Investigational	OPRM1	Appetite regulation	(130)
Melatonin	Sleep disorders	Investigational	MTNR1A, MTNR1B	Circadian glucose regulation	(131)
Niclosamide	Anthelmintic	Preclinical	Multiple mitochondrial targets	AMPK activation	(132)
Berberine	Herbal antimicrobial	Clinical studies	AMPK	AMPK activation	(133)
Resveratrol	Nutraceutical	Investigational	SIRT1	SIRT1–AMPK axis	(134)
Curcumin	Anti-inflammatory	Clinical studies	NFKB1, NRF2	NF-κB inhibition	(135)

Overview of selected drugs, their original clinical indications, and current status in diabetes research or therapy, highlighting the corresponding primary molecular targets (genes/proteins) and key signaling pathways involved.

Notably, the second functional cluster of oGPCRs shares several functional characteristics with GLP1R-targeted therapies in regulating metabolism and reducing chronic inflammation associated with diabetes (87). Among this group, GPR21 acts as a key proinflammatory mediator. Genetic deletion of GPR21 has been shown to improve glucose tolerance and insulin sensitivity, demonstrating its important role in metabolic inflammation (19). These effects closely resemble the anti-inflammatory actions of GLP1R agonists, which suppress pro-inflammatory cytokines, such as TNF-α and IL-1β. Several receptors within this cluster also participate in the central appetite regulation and hypothalamic signaling. These functions are similar to the central mechanisms through which GLP1R therapies coordinate energy homeostasis, metabolic regulation, and systemic immune responses via neuroimmune crosstalk. In addition, many of these receptors influence intracellular signaling pathways that converge on the cAMP-PKA axis, which is a major downstream pathway of GLP1R activation. This signaling axis regulates NF-κB activation, oxidative stress, and inflammation. It may also modulate inflammation associated with 20-HETE signaling via GPR75 (136). Overall, the oGPCR cluster represents a promising yet largely unexplored therapeutic target. Targeting these receptors may reproduce or complement the anti-inflammatory and metabolic benefits of incretin-based therapy. Such strategies could provide new opportunities to alleviate chronic metabolic inflammation and slow the progression of diabetes and its associated complications in humans.

GLP1R therapies, such as semaglutide and liraglutide, effectively reduce chronic low-grade inflammation associated with T2D by targeting the metabolic, oxidative, and inflammatory pathways that drive disease progression. These therapies primarily enhance the cAMP-PKA signaling pathway. This signaling suppresses NF-κB activation and reduces the production of proinflammatory cytokines, including TNF-α, IL-6, and IL-1β (137). GLP1R agonists improve glycemic control while simultaneously alleviating systemic inflammation and insulin resistance. This therapeutic paradigm is highly relevant to several functional clusters of oGPCRs that share similar physiological roles and intracellular signaling pathways. For example, secretagogue receptors, such as GPR119 and GPR142, functionally overlap with the GLP1R pathway by activating the same Gs-cAMP-PKA signaling cascade to regulate glucose homeostasis and inflammatory responses. Similarly, metabolite-sensing receptors, including HCAR2 and GPR84, connect lipid-derived metabolites to AMPK activation and suppression of NF-κB-mediated inflammatory signaling (137). Receptors within the second functional cluster, such as GPR21 and GPR75, also contribute to neuro-metabolic integration and central appetite regulation (19). These functions closely resemble the mechanisms through which incretin-based therapies coordinate energy homeostasis, metabolic regulation and immune responses. These findings suggest that targeting selected oGPCRs may complement or enhance the therapeutic benefits of GLP1R agonists. Therefore, oGPCR networks represent promising yet largely unexplored therapeutic targets for reducing chronic inflammation, restoring metabolic homeostasis, and preventing the progression of diabetes and its associated complications in humans.

## 9 Repurposing non-diabetes drugs for diabetes

Drug repurposing for diabetes can be rationalized by profiling existing pharmacological agents on orphan GPCRs that regulate diabetes-related signaling. Several receptors, such as GPR35, GPR55, GPR84, GPR39, and GPR146, are important signaling receptors that link fat sensing, immune responses, and β-cell function. These signaling pathways overlap with established antidiabetic mechanisms, such as incretin signaling, AMPK activation, and inflammatory modulation. This provides opportunities for repurposing existing drugs as ligands to modulate these receptors, either directly or indirectly, for the treatment of diabetes (**Table 1**).

Notably, GPR35 is a Gi-coupled receptor that is activated by kynurenic acid and inflammatory metabolites (59). Its activation reduces cAMP levels and modulates macrophage signaling. This suggests that anti-inflammatory drugs or modulators of the tryptophan pathway could be repurposed to attenuate insulin resistance in patients with obesity. Similarly, GPR84 is a proinflammatory receptor activated by medium-chain fatty acids (82). It is highly expressed in the immune cells. Targeting this receptor using anti-inflammatory or lipid-modifying agents may suppress NF-κB and TNF-driven inflammation, thereby improving insulin sensitivity. Metabolic GPCRs, such as GPR39 and GPR55, directly influence β-cell function and glucose sensing. GPR39 is a zinc-responsive receptor that enhances insulin secretion via Gq-mediated calcium signaling. This indicates that zinc-modulating compounds may improve β-cell responsiveness (50). GPR55 links endocannabinoid signaling to insulin secretion and energy balance (43). This suggests the potential repurposing of lipid-signaling modulators to restore metabolic homeostasis. GPR146 is a cholesterol-responsive receptor involved in lipid-glucose cross-talk (46). Lipid-lowering agents, such as statins or bile acid modulators, may indirectly influence their activity and improve glucose metabolism (138). Adhesion GPCRs, such as ADGRF5 and GPR56, regulate endothelial integrity, vascular inflammation, and tissue remodeling. Drugs targeting the RAAS, PPARγ, or SGLT2 pathways may indirectly converge on these receptors by restoring PI3K-AKT signaling and improving vascular and metabolic homeostasis.

Recent systems pharmacology and transcriptomic studies suggest that diabetes involves not only classical insulin signaling but also the broader dysregulation of GPCR networks and orphan receptors. For example, GPR50 deficiency increases adipose inflammation and inhibits insulin signaling (32), whereas GPR26 downregulation is associated with oxidative stress under hyperglycemic conditions (139). In addition, GPR135, GPR22, and GPR162 show tissue-specific dysregulation in the diabetic heart, kidney, and brain, suggesting their crucial roles in diabetes (140). In addition, clomiphene citrate improves insulin and glucose parameters in insulin-resistant patients (141), and LY294002, a PI3K inhibitor, can regulate the PI3K-AKT pathway in insulin signaling (142). These findings suggest that diabetes is driven by the complex interactions of many signaling pathways, including GPCR signaling, immune responses, and kinase-regulated pathways. This provides opportunities to develop novel therapeutics by targeting oGPCRs and repurposing existing drugs.

In addition, several non-diabetic drugs have been validated for improving glycemic control and managing diabetes-related complications (**Table 1**). Among these, bromocriptine, a dopamine D2 receptor agonist initially approved for Parkinson’s disease, has been repurposed for T2D and is FDA-approved for the improvement of glycemic control (101). In addition, pregabalin, originally developed as an antiepileptic agent, is widely used to improve diabetic neuropathic pain. Calcium channel blockers and angiotensin-converting enzyme inhibitors have also shown potential glucose-lowering effects in patients with diabetes. Similarly, α1-adrenoceptor antagonists, commonly used for benign prostatic hyperplasia (143), have been found to improve blood glucose control. Salicylates, including aspirin and salsalate, exhibit antidiabetic effects by suppressing inflammation-associated insulin resistance pathways (144). Similarly, hydroxychloroquine, which is widely used to treat malaria and autoimmune diseases, has beneficial effects on insulin sensitivity and pancreatic β-cell function (145). These findings highlight the potential of repurposing existing drugs to expand the therapeutic options for T2D. In addition, we highlight some plant-derived compounds obtained from various medicinal plants that may interact with specific oGPCR targets and/or their downstream signaling pathways to regulate metabolic processes, thereby showing potential therapeutic relevance in the management of diabetes and related metabolic disorders (**Table 2**).

**Table 2:** Bioactive compounds and their possible oGPCR and signaling pathways in the regulation of metabolic and diabetic disorders.

Plant-derived compound(s)	Plant source	Receptor	Key signaling pathway	Metabolic & diabetic relevance	References
Oleoylethanolamide (OEA)	Olive oil-associated diet (*Olea europaea*)	GPR119	Gαs → cAMP → GLP-1 secretion	Strong antidiabetic and anti-obesity effects; β-cell protection	(146)
Cannabidiol (CBD), CBG	*Cannabis sativa*	GPR55	ERK1/2, RhoA, Ca^2+^	Regulates obesity and insulin signaling; anti-inflammatory potential	(147)
Quercetin, Kaempferol	Fruits, vegetables, tea	GPR35	Gi/o, ERK1/2, β-arrestin	Involved in glucose regulation, obesity-related inflammation, and nephropathy	(148)
Puerarin, flavonoids	*Pueraria lobata*	GPR39	PI3K/Akt, ERK, CREB	Promotes β-cell survival, glucose control, and diabetic wound healing	(149)
Fiber-derived metabolites, polyphenol-rich diets	Whole grains, legumes	HCAR2	Gi → anti-inflammatory	Improves insulin sensitivity; protects against retinopathy and nephropathy	(150)
Hydroxy-fatty acid metabolites	Plant oils	HCAR3	Gi signaling	Emerging role in glucose and lipid metabolism	(151)
Medium-chain fatty acids	Coconut oil, palm kernel oil	GPR84	ERK, NF-κB	Associated with obesity-driven inflammation and insulin resistance	(56)
Oxysterol-modulating phytosterols (indirect)	Plant sterols from nuts, seeds, vegetable oils	GPR183 (EBI2)	Gi → ERK, Akt	Linked to insulin resistance, adipose inflammation, and diabetic inflammation	(152)
Polyphenol-rich extracts (indirect modulation reported)	Green tea, berries, grapes	GPR21	MAPK, insulin signaling pathways	Regulates insulin sensitivity and obesity-associated metabolic syndrome	(153)
Flavonoid-rich phytochemicals (indirect evidence)	Tea, citrus fruits	GPR61	cAMP signaling	Potential role in energy balance and metabolic regulation	(154)
Polyunsaturated lipid-derived phytochemicals	Plant seed oils	GPR12	cAMP, neurite signaling	Involved in energy expenditure and feeding behavior	(155)
Curcumin and anti-inflammatory phytochemicals (indirect pathway modulation)	*Curcuma longa*	GPR56 (ADGRG1)	RhoA, PI3K/Akt, ERK	Associated with adipose remodeling and vascular protection	(156)
20-HETE pathway modulators and polyphenols (indirect)	Various medicinal plants	GPR75	PI3K/Akt, ERK	Obesity-linked receptor with potential glucose and cardiovascular benefits	(157)
Polyphenols affecting succinate metabolism	Green tea, berries, grapes	GPR91 (SUCNR1)	ERK1/2, Ca^2+^, MAPK	Implicated in diabetic nephropathy, retinopathy, and adipose inflammation	(158)
Flavonoids and amino acid metabolism modulators (indirect)	Soy, legumes	GPR142	Gq → insulin secretion pathways	Supports β-cell function and glucose homeostasis	(81)

This table summarizes plant-derived bioactive compounds, their natural plant sources, and their molecular targets, including receptor interactions and downstream signaling pathways. This highlights their potential roles in regulating metabolic processes and their relevance to diabetes and related metabolic disorders.

## 10 Challenges and limitations in orphan GPCR research and therapeutic translation

Despite the considerable therapeutic potential of oGPCRs, several critical challenges continue to delay their translation into clinical applications. One of the main challenges is the uncertainty in receptor deorphanization because many proposed ligand-receptor pairings remain unconfirmed or have failed to be reproduced in independent studies. This uncertainty is further exacerbated by species-specific signaling difference nondiabeticologous receptors exhibit substantial pharmacological variability. For example, GPR35 displays up to a 100-fold difference in ligand potency between rodents and humans (159). Another major challenge is ligand and system bias. Receptor signaling outcomes are highly dependent on cellular context, transducer stoichiometry, receptor expression levels, and experimental conditions (160). Consequently, pharmacological responses observed in recombinant overexpression systems often fail to accurately predict receptor behavior in native human tissues. Functional characterization is further complicated by the highly tissue-specific and spatially regulated expression of GPCRs (161). The same receptor can mediate distinct physiological or pathological effects depending on the cell type, tissue, or subcellular compartment in which it is expressed. Moreover, robust experimental validation remains limited for most oGPCRs, as most mechanistic evidence is derived from animal models or engineered cell systems that may not faithfully recapitulate human physiology (162). Overall, these limitations create a significant translational gap between mechanistic discoveries and the development of clinically effective oGPCR-targeted therapy. Bridging this translational gap requires reliable receptor deorphanization and validation using physiologically relevant human models. In addition, improved methods are required to identify endogenous ligands. These ligands are often transiently produced, present at extremely low concentrations, or expressed only under specific physiological or pathological conditions of the body.

## 11 Future directions

The exponential growth and mortality rates of patients with diabetes are alarming and highlight the need to change the future direction of efforts aimed at reducing the global burden of diabetes. Among several advanced technologies, high-resolution omics technologies combined with computational models have provided better therapeutic opportunities for the management of diabetes. This multidisciplinary approach has facilitated the deorphanization of many GPCRs by identifying their endogenous ligands and elucidating their functions. These receptors have also emerged as crucial biomarkers and therapeutic targets for many diseases. Several oGPCRs are expressed in pancreatic islets, immune cells, adipose tissue, and the gastrointestinal tract, where they regulate insulin secretion, glucagon release, β-cell survival, inflammation, and glucose homeostasis.

Single-cell RNA sequencing (scRNA-seq) has also provided an opportunity to study the cellular heterogeneity of individual pancreatic islet cells. This technology has enabled the identification of rare cell populations involved in autoimmune processes. scRNA-seq has also shown that GPR119 is expressed at approximately 10-fold higher levels in human glucagon-producing α-cells than in β-cells, suggesting a more complex role in regulating the glucagon-insulin axis (163). Thus, single-cell technology may provide deeper insights into cell-specific oGPCRs for deorphanization and their roles in diabetes treatment.

In addition to scRNA-seq, spatial transcriptomics (ST) allows in-depth gene expression profiling of the islet microenvironment and monitoring of cellular interactions (163). Recent advances in ST, including deep generative models such as SpatialScope, have improved the ability to understand the single-cell spatial distributions (163). Furthermore, ST of oGPCR expression within islets and immune cell niches may provide better insights into receptor-mediated signaling networks involved in disease progression and therapeutic response.

Notably, advancements in artificial intelligence (AI)-guided ligand prediction have accelerated the drug development process. Specifically, machine learning approaches, such as deep neural networks (DNNs) and quantitative structure-activity relationship (QSAR) models, allow the screening of large numbers of compounds or drugs (164). The oGPCR structure-based identification of receptor-specific compounds allowed for the investigation of the mechanisms of action of new ligands. Recently developed CatBoost algorithms have been shown to predict several active compounds with an accuracy of over 90%, thereby accelerating the drug development process (165). In addition, the structure-based drug design (SBDD) approach allows the development of knowledge graphs that integrate multimodal datasets and cryo-electron microscopy (cryo-EM) structures (166). This integration facilitates the identification of potential repurposed drugs and accelerates the development of targeted therapies. Furthermore, this will allow us to identify selective GPCR modulators that target disease-specific pathways.

Overall, these technologies may provide opportunities for developing precision medicine for the treatment of diabetes. For instance, an analysis of scRNA-seq-derived metagenomics found a link between T1D and genetic variants of Melatonin Receptor 1B (MTNR1B) (37). This allows clinicians to predict disease progression and may be a therapeutic target for treating diabetes. Similarly, patient-specific molecular profiling of islet cells may facilitate the development of oGPCR-targeted therapies. Thus, the deorphanization of oGPCRs may provide potential therapeutic targets for developing patient-specific treatments based on the molecular subtypes of diabetes. Overall, oGPCRs may offer promising therapeutic opportunities for advancing precision medicine in diabetes treatment.

## 12 Conclusion

In conclusion, oGPCRs play a crucial role in diabetes and are one of the largest underexplored molecular targets. A systematic evaluation of the biological functions of oGPCRs, deorphanization status, and drug repurposing potential has identified several receptors with clinical potential. These receptors regulate crucial metabolic processes in the pancreas, adipose tissue, liver, and central nervous system (CNS). Specifically, they regulate several cellular processes involved in insulin secretion, nutrient sensing, inflammation and energy homeostasis. This review highlights the therapeutic potential of oGPCRs by classifying them into key functional groups involved in the pathophysiology of diabetes. These include secretagogues, such as GPR119 and GPR142; regulatory modulators, such as GPR27; and metabolic sensors, such as GPR75 and GPR91. In addition, drug repurposing strategies have identified potential pathway targets for the development of novel therapeutics for diabetes. As key regulators of metabolic, immune, and vascular pathways, oGPCRs may be potential targets for repurposable drugs for diabetes treatment. Recent scRNA-seq, spatial transcriptomics, AI-driven ligand discovery, and structure-based drug design technologies are accelerating GPCR deorphanization, which may provide new opportunities to screen novel therapeutics for diabetes. Overall, oGPCR deorphanization holds significant potential for improving diabetes therapy through novel target discovery, drug repurposing, and precision medicine.

## Glossary

*GPCR*: G-protein-coupled receptors
oGPCRs: orphan G-protein-coupled receptors
aGPCR: adhesion G-protein-coupled receptor
GAIN: GPCR autoproteolysis-inducing domain
GPS: GPCR proteolysis site
ECRs: N-terminal extracellular regions
ECL2: extracellular loop 2
HCARs: hydroxy-carboxylic acid receptors
CELSR: cadherin EGF LAG seven-pass G-type receptor
mGlyR: metabotropic glycine receptor
T1D: type 1 diabetes
T2D: type 2 diabetes
MODY: maturity-onset diabetes of the young
IR: insulin resistance
BMI: body mass index
NAFLD: non-alcoholic fatty liver disease
MAFLD: metabolic dysfunction-associated fatty liver disease
MASH: metabolic dysfunction-associated steatohepatitis
GLP-1: glucagon-like peptide-1
GIP: glucose-dependent insulinotropic polypeptide
cAMP: cyclic adenosine monophosphate
GSIS: glucose-stimulated insulin secretion
CART: cocaine- and amphetamine-regulated transcript
LPC: lysophosphatidylcholine
LPI: lysophosphatidylinositol
LPA: lysophosphatidic acid
MCFAs: medium-chain fatty acids
FFAs: free fatty acids
20-HETE: 20-hydroxyeicosatetraenoic acid
BHB: β-hydroxybutyrate
TNF-α: tumor necrosis factor-alpha
IL-1β: interleukin-1 beta
IL-6: interleukin-6
BigLEN: a proSAAS-derived neuropeptide
RANTES: regulated upon activation, normal T cell expressed and secreted
AMPK: adenosine monophosphate-activated protein kinase
SGLT2: sodium-glucose cotransporter 2
DPP-4: dipeptidyl peptidase-4
PLC: phospholipase C
MAPK: mitogen-activated protein kinase
JNK: c-Jun N-terminal kinase
PKA: protein kinase A
PKC: protein kinase C
IRS1: insulin receptor substrate 1
ACE: angiotensin-converting enzyme
RAAS: renin-angiotensin-aldosterone system
TCA cycle: tricarboxylic acid cycle
PPARγ: peroxisome proliferator-activated receptor gamma
PEPCK: phosphoenolpyruvate carboxykinase
G6Pase: glucose-6-phosphatase
scRNA-seq: single-cell RNA sequencing
ST: spatial transcriptomics
AI: artificial intelligence
DNNs: deep neural networks
QSAR: quantitative structure-activity relationship
SBDD: structure-based drug design
cryo-EM: cryo-electron microscopy
HFD: high-fat diet
RER: respiratory exchange ratio
PCP: planar cell polarity
POMC: proopiomelanocortin
BDNF: *brain-derived neurotrophic factor.*
